# Inhibition of BK_Ca_ negatively alters cardiovascular function

**DOI:** 10.14814/phy2.13748

**Published:** 2018-06-21

**Authors:** Nishi H. Patel, Justin Johannesen, Kajol Shah, Sumanta K. Goswami, Neel J. Patel, Devasena Ponnalagu, Andrew R. Kohut, Harpreet Singh

**Affiliations:** ^1^ Department of Internal Medicine Drexel University College of Medicine Philadelphia Pennsylvania; ^2^ Department of Pharmacology and Physiology Drexel University College of Medicine Philadelphia Pennsylvania; ^3^ Penn Heart and Vascular Center University of Pennsylvania Philadelphia Pennsylvania; ^4^ Division of Cardiology Drexel University College of Medicine Philadelphia Pennsylvania

**Keywords:** BK_C__a_ channels, cardiac function, echocardiography, paxilline

## Abstract

Large conductance calcium and voltage‐activated potassium channels (BK_C__a_) are transmembrane proteins, ubiquitously expressed in the majority of organs, and play an active role in regulating cellular physiology. In the heart, BK_C__a_ channels are known to play a role in regulating the heart rate and protect it from ischemia–reperfusion injury. In vascular smooth muscle cells, the opening of BK_C__a_ channels results in membrane hyperpolarization which eventually results in vasodilation mediated by a reduction in Ca^2+^ influx due to the closure of voltage‐dependent Ca^2+^ channels. Ex vivo studies have shown that BK_C__a_ channels play an active role in the regulation of the function of the majority of blood vessels. However, in vivo role of BK_C__a_ channels in cardiovascular function is not completely deciphered. Here, we have evaluated the rapid in vivo role of BK_C__a_ channels in regulating the cardiovascular function by using two well‐established, rapid‐acting, potent blockers, paxilline and iberiotoxin. Our results show that BK_C__a_ channels are actively involved in regulating the heart rate, the function of the left and right heart as well as major vessels. We also found that the effect on BK_C__a_ channels by blockers is completely reversible, and hence, BK_C__a_ channels can be exploited as potential targets for clinical applications for modulating heart rate and cardiac contractility.

## Introduction

BK_Ca_ channels are voltage and calcium‐activated potassium channels found in a multitude of cells throughout the body, including vascular smooth muscle cells (VSMCs) and neurons in which they function to regulate cell tone and excitability (Singh et al. 2012; Toro et al. 2014; Balderas et al. 2015). On intracellular injections of Ca^2+^, Meech in 1970s reported an increase in K^+^ conductance in nerve cells which set the foundation for the existence of BK_Ca_ channels (Brown et al. 1970). The opening of BK_Ca_ channels results in fast repolarization of the cellular membrane and hence, closure of voltage‐dependent Ca^2+^ channels, resulting in reduced Ca^2+^ entry into the cell and increased Ca^2+^ extrusion by the Na^+^–Ca^2+^ exchanger. Therefore, the primary function of BK_Ca_ is to exert a negative feedback on the membrane potential and on intracellular Ca^2+^ (Toro et al. 2014).

BK_Ca_ channels are heterogeneously expressed in the cardiovascular system (Singh et al. 2012; Balderas et al. 2015). The pore‐forming *α*‐subunit encoded by a gene, *Kcnma1*, is present in the membranes of many cell types, except adult cardiomyocytes where they are exclusively present in mitochondria (Xu et al. 2002; Singh et al. 2013; Soltysinska et al. 2014; Toro et al. 2014). The regulatory *β* subunit (encoded by four genes, *Kcnmb1‐4*) is expressed in the heart and vascular smooth muscle cells. A recently identified *γ* subunit (LRRC26) is also present in cerebral artery smooth muscle cells (Evanson et al. 2014). BK_Ca_ channels are considered key players in the vascular system, where they play a major role in the regulation of vascular tone. During depolarization of VSMCs, BK_Ca_ channels open to guard against excessive vasoconstriction. Ex vivo experiments revealed that blockage of BK_Ca_ channels with pharmacologic agents results in aortic and carotid artery constriction. In cardioprotection from ischemia–reperfusion studies, activation of BK_Ca_ channels results in a reduction in myocardial infarction, whereas blocking by Paxilline (PAX) ablated cardioprotection from ischemic preconditioning (Singh et al. 2013; Toro et al. 2014; Balderas et al. 2015).

Over the last decade, significant progress has been made to understand the role of BK_Ca_ channels in cardiac function. The *α*‐subunit of the channel has been shown to be involved in regulation of heart rate through the sinoatrial (SA) nodal cell regulation (Imlach et al. 2010; Lai et al. 2014). However, the role of BK_Ca_ channels in the regulation of cardiac function is not deciphered. In order to understand the role of BK_Ca_ channels in cardiac and vascular function of major vessels, we have used a combination of pharmacology and echocardiography. Using a rat model, we have injected either PAX (cell permeable) or iberiotoxin (IBTX, cell impermeable) into the left femoral vein. In this study, we have used comprehensive 2D echocardiography to evaluate the effects of the highly specific inhibitors PAX and IBTX on several physiologic parameters of left and right ventricular function (Gao et al. 2000, 2011; Kohut et al. 2016), as well as the hemodynamic status of the cardiovascular system. Echocardiography images were acquired under anesthesia, and various parameters of cardiovascular function were evaluated.

We have found that blocking BK_Ca_ channels reversibly alters the cardiovascular function by reducing the heart rate and increases contraction of major vessels. We also found that the inhibitory effect of BK_Ca_ is completely reversible. Our study is the first in vivo comprehensive study to establish the role of BK_Ca_ channels in cardiovascular function.

## Methods

### Animals

In this study, 2‐month‐old Sprague–Dawley male rats (Charles River Laboratories, Wilmington, MA) weighing 300–330 g were used for experiments. The experimental procedures were designed in accordance with National Institutes of Health and American Association for the Accreditation of Laboratory Animal Care (AAALAC) guidelines and approved by the Drexel University College of Medicine Institutional Animal Care and Use Committee (IACUC), Philadelphia, PA.

### Cardiovascular function analysis

A high‐frequency, high‐resolution digital imaging platform with linear array technology and Color Doppler Mode for in vivo high‐resolution micro‐imaging was used for echocardiography as per previously published guidelines (Kohut et al. 2016) (Vevo^®^ 2100 Imaging System, FUJIFILM VisualSonics Inc., Toronto, Canada). For assessing the cardiovascular function of rats, a high‐frequency transducer probe (VisualSonics MS250 with a frequency range of 18–38 MHz) was utilized as it provides appropriate resolution and depth of penetration needed.

Anesthesia was achieved using 2.5% (*v/v*) isoflurane mixed with oxygen. Rats were evaluated with a toe pinch to ensure a complete anesthesia. After achieving an adequate anesthesia, anthropomorphic measurements were taken. Total body weight and nose to anus length were recorded, and rats were secured on a prewarmed (37°C) imaging platform. Hairs from the ventral chest were removed using commercially available hair removal cream (Nair™, © Church & Dwight Co). After securing each rat to the imaging platform, anesthesia concentration was titrated from 1% to 3% to maintain a minimum heart rate of 300 beats/min.

Baseline echocardiograms were obtained for each rat. After baseline echocardiogram, anesthesia was maintained, and the left femoral vein of each rat was dissected and exposed. Using a 23‐gage needle and 1‐mL syringe, rats were injected with 1 mL/kg of a solution of PAX (final concentrations, 5.7 and 115 nmol/L) or IBTX (final concentrations, 1 and 10 *μ*mol/L), and DMSO (control). An echocardiogram was repeated at 1 and 15 min after injection. Mean arterial pressure was measured by Miller transducer inserted into the carotid. A total of 4–6 rats were assigned to each group. The total anesthesia time for each animal did not exceed 25 min, with each echocardiogram taking 5–10 min to complete.

### Image analysis

All echocardiograms were reviewed using the VisualSonic Vevo Lab software in conjunction with a clinical echocardiographer to ensure optimal image quality and analysis.

**Figure 1:**
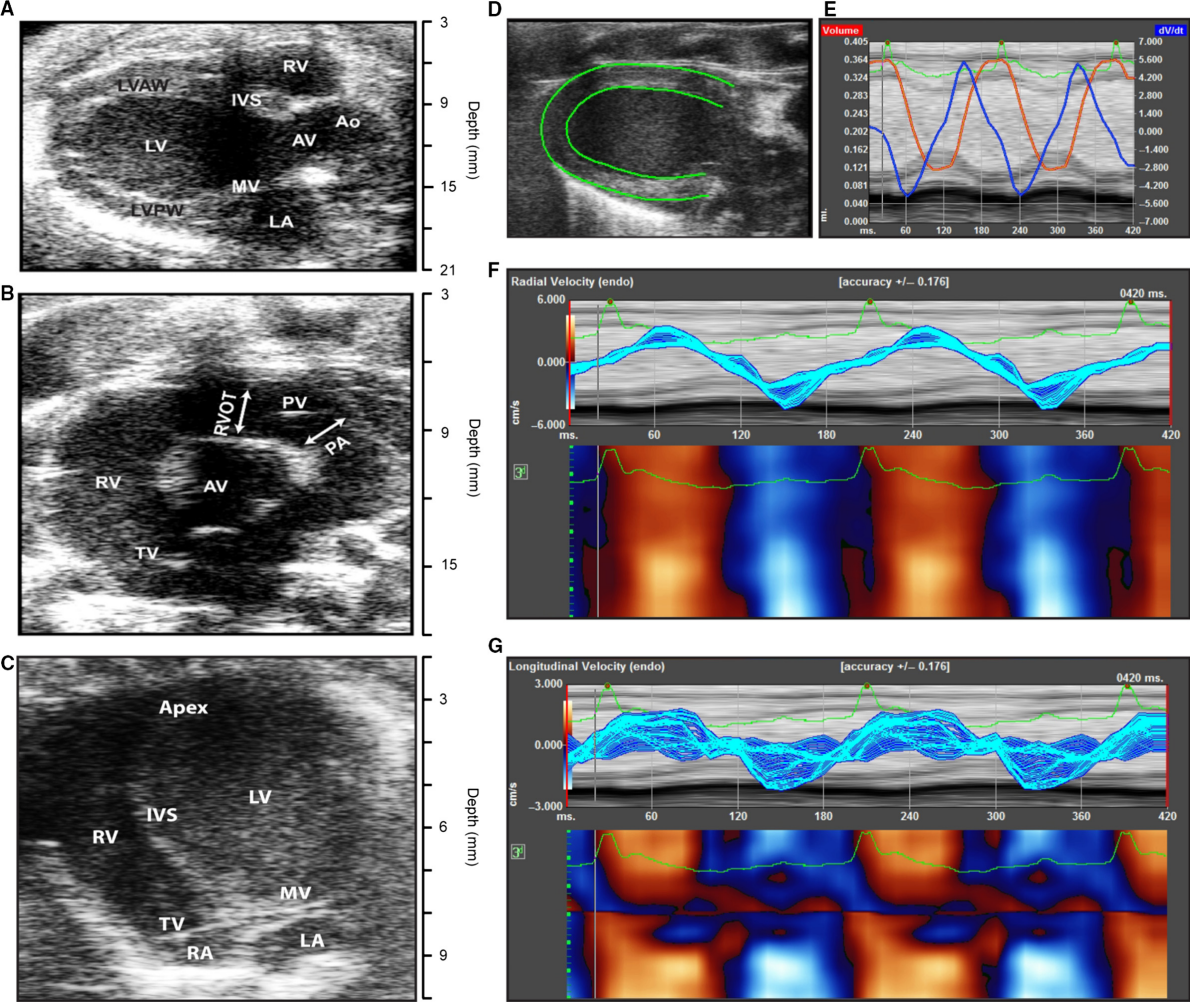
Echocardiographic analysis of the heart. (A) Parasternal long‐axis view of the heart. (B) Short‐axis view of the heart. (C) Apical four‐chamber view of the heart. (D) Tracing the left ventricular myocardial borders for strain analysis. (E) Strain analysis indicating left ventricular volume (red) and derivative of tissue velocity (blue) wall motion in two cardiac cycles. The green line indicates the corresponding EKG. (F) Radial strain for two long‐axis LV contractions. (G) Longitudinal strain for two long‐axis LV contractions. Ao, aorta; AV, aortic valve; IVS, interventricular septum; LA, left atrium; LV, left ventricle; LVAW, left ventricular anterior wall; LVPW, left ventricular posterior wall; MV, mitral valve; PA, pulmonary artery; PV, pulmonic valve; RA, right atrium; RV, right ventricle; RVOT, right ventricular outflow tract; TV, tricuspid valve.

Specific views were captured using various echo imaging modalities to obtain the physiologic parameters of interest (Gao et al. 2000, 2011; Kohut et al. 2016). Figure 1 shows B mode views of the rat heart obtained during 2D echocardiography. Figure 1A shows the parasternal long‐axis view of the heart acquired using B mode. From this view, left ventricle (LV) functional parameters were measured including LV ejection fraction (LVEF). In addition, the postimaging analysis included LV strain analysis of the parasternal long‐axis (PSLAX) views to calculate Global Longitudinal Strain (GLS). Figure 1B shows the parasternal short‐axis (PSSAX) view at the level of the aortic valve toward the base of the heart. The pulmonic valve and tricuspid valve are also seen here, and this view is ideal for assessing right ventricular outflow tract and pulmonary artery diameter. Doppler echocardiography is also obtained from this view with sample volume in the pulmonary artery at the valve leaflet tips. Figure 1C shows the apical four‐chamber view. Other parameters of LV systolic function, such as Mitral Annular Plane Systolic Excursion (MAPSE) and Tissue Doppler imaging, can be obtained by placing the sample volume at the lateral mitral annulus and angulating the M‐Mode or Doppler plane along the red line.

### Statistical analysis

For physiologic parameters of interest, four measurements for each animal were taken and averaged. Notably, at high doses of PAX, several animals experienced cardiac arrest prior to the 15‐min time interval. Hemodynamic data at the 15‐min interval was assigned a value corresponding to 1% of the baseline to avoid calculations involving null values.

All values were entered into Microsoft Excel, and Student's *t*‐test was used to compare means for statistical significance. All values are reported as mean with standard error.

## Results

### Inhibition of BK_Ca_ causes bradycardia

**Figure 2:**
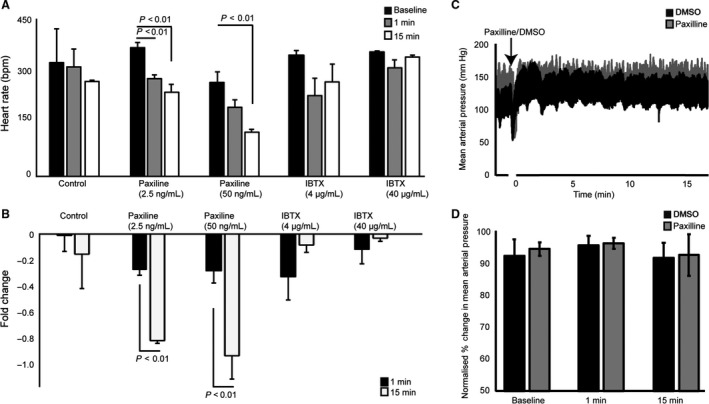
Change in heart rate (beats per minute) after administration of Paxilline or Iberiotoxin. (A) Absolute reduction in heart rate after administration of DMSO control compared with low‐dose and high‐dose Paxilline or Iberiotoxin at baseline (*n* = 5–7), 1 min, and 15 min after injection. (B) Fold change in heart rate from baseline for the three groups at 1 min and 15 min after injection. Treatment with Paxilline at low dose resulted in decreased heart rate at 1 and 15 min. At high doses, a decrease in heart rate was also seen. Treatment with the higher dose of Paxilline showed a greater fold reduction in heart rate at 15 min compared with the low‐dose Paxilline. The use of Iberiotoxin did not significantly affect the heart rate. (C) Representative mean arterial pressure traces with or without (50 ng/mL Paxilline, *n* = 4). (D) Percentage change of MAP normalized to MAP before Paxilline injection. There was no difference observed in the MAP with Paxilline as compared with the DMSO control.

Inhibition of BK_Ca_ channels with PAX and lolitrem B is known to reduce heart rate in wild‐type but not in *Kcnma1* ^*−/−*^ mice (Imlach et al. 2010). Ex vivo studies in rat hearts also showed a reduction in heart rate when perfused with PAX and lolitrem B. We tested the role of BK_Ca_ in the regulation of heart rate in in vivo model. Heart rate was measured at both 1‐ and 15‐min intervals after injecting PAX and IBTX. As mentioned previously, prior studies have noted the negative chronotropic effect of BK_Ca_ channel inhibition on heart rate and have also elucidated the mechanism of this relative bradycardia via an effect on SA nodal cells (Lai et al. 2014). Both high‐ (50 ng/mL) and low‐dose (2.5 ng/mL) PAX groups experienced significant reductions in heart rate in rats (Fig. 2).

Immediately after injection of PAX (after 1 min) both 2.5‐ and 50‐ng/mL groups showed a ~1–25% reduction in heart rate (Fig. 2), but animals injected with 50 ng/mL showed ~40–50% reduction and was fatal in over 50% animals injected with PAX. Surprisingly, IBTX showed no change in heart rate after 1 or 15 min at lower (4 *μ*g/mL) or higher dose (40 *μ*g/mL) (Fig. 2). To test whether the reduction in heart rate is mediated by a change in blood pressure, we measured mean arterial pressure after Paxilline (5 ng/mL). As shown by other groups, we did not detect any changes in the MAP within 1 or 15 min of Paxilline injection (Imlach et al. 2010). Our results agree with ex vivo and knock out studies showing the role of BK_Ca_ in regulating the heart rate and also provided us valuable pharmacological tools to evaluate cardiovascular function in vivo.

### Inhibition of BK_Ca_ causes left ventricular dysfunction

**Figure 3:**
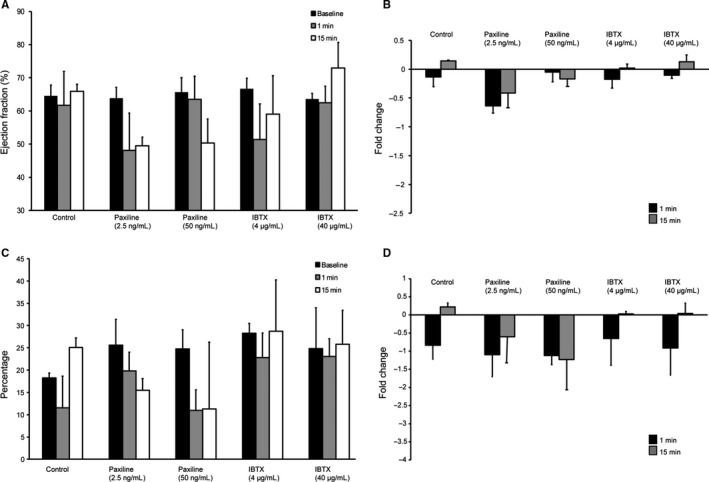
Evaluation of left ventricular function after administration of Paxilline (*n* = 7) or Iberiotoxin (*n* = 4). (A) Bar graphs showing an absolute reduction in left ventricular ejection fraction. (B) Fold change in left ventricular ejection fraction obtained from A. (C) Bar graph showing changes in absolute global longitudinal strain values. (D) Fold change in global longitudinal strain calculated from C. No statistically significant reduction in left ventricular ejection fraction was found. Although not statistically significant, strain analysis revealed a trend toward reduction in left ventricular function seen with Paxilline at low and high doses at both 1 and 15 min after drug administration.

We performed a comprehensive analysis of the LV of rats after injecting DMSO (control), PAX, or IBTX. Changes in LVEF in response to DMSO, PAX, and IBTX were measured using the PSLAX view. DMSO had no impact on the cardiac ejection fraction. As shown in Figure 3A and B, there were no significant changes in the LVEF with control, PAX, or IBTX. PAX at 15 min showed a small but not statistically significant reduction in LVEF. Qualitatively, injection of PAX did induce abnormalities in wall motions.

To analyze the LV function, we also performed global left ventricle strain analysis (Figs. 1D–G and 3C, and D). Myocardial strain analysis allows for detection of more subtle changes in LV function than an assessment of LVEF alone (Figs. 3C and D). In agreement with LVEF, strain analysis did not yield any significant differences between control or experimental (PAX or IBTX) animals. As observed for LVEF, LV strain also showed reduction with PAX, but it was not statistically significant. In a null mutant mouse, a small reduction in LVEF (from ~60% to ~55%) was reported (Frankenreiter et al. 2017). However, a slight reduction in LV function with PAX and wall movement abnormalities indicated a possible dysfunction of coronary arteries which we tested further in detail.

### Effect of BK_Ca_ inhibition on coronary artery flow

**Figure 4:**
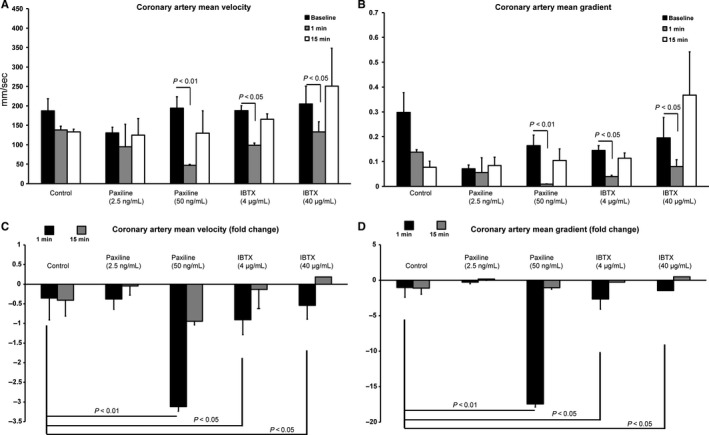
Evaluation of coronary flow after administration of Paxilline (*n* = 5) or Iberiotoxin (*n* = 4). (A) Absolute reduction in mean coronary velocity. (B) Absolute reduction in mean coronary gradient. (C) Fold change in mean coronary velocity. (D) Fold change in mean coronary gradient. Velocity through the left main coronary artery was found to be decreased 1 min after administration of high‐dose Paxilline. This reduction in flow normalized at 15 min. Mean coronary gradient in this particular group was also found to be decreased.

BK_Ca_ channels are abundantly expressed in the plasma membrane of coronary artery SMC where they are predicted to play a role in regulating coronary flow. They are known to play an active role in protecting the heart from IR injury (Ahn et al. 1994). However, most of the studies are carried out in isolated cells, vessels, or heart. For the first time, we performed in vivo analysis of the coronary artery flow by using PAX and IBTX (Fig. 4). Color Doppler movies were acquired and flow rates were measured. We found that inhibition of BK_Ca_ by PAX significantly (*P* < 0.01) reduced mean coronary velocity and coronary mean gradient at 50 ng of PAX within 1 min but was completely reversible after 15 min (Fig. 4). Similarly, IBTX showed a significant (*P* < 0.05) reduction in the mean velocity and mean gradient (Fig. 4) at 4 as well as 40 *μ*g/mL which completely reversed within 15 min. These results implicate BK_Ca_ channels in regulating the coronary perfusion.

### Effect of BK_Ca_ inhibition on pulmonary function

**Figure 5:**
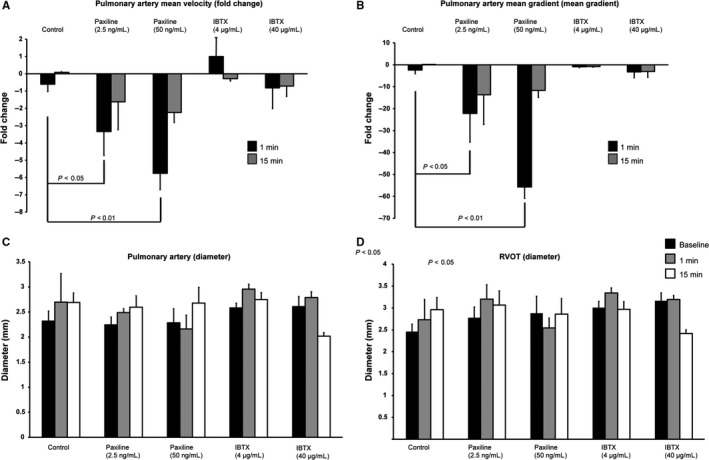
Evaluation of pulmonic artery and right heart function after administration of Paxilline (*n* = 5) or Iberiotoxin (*n* = 4). (A) Bar graph showing fold change in mean pulmonary velocity. (B) Bar graph representing fold change in mean pulmonary gradient. (C) Absolute change in pulmonary artery diameter. (D) Absolute change in right ventricular outflow tract diameter. Velocity across the pulmonic valve was decreased 1 min after administration of both low‐dose and high‐dose Paxilline. In both dosages, velocity began to show normalization by 15 min. Mean pulmonary gradient showed a similar trend. In both cases, Iberiotoxin did not show an effect. No differences were seen in pulmonary artery diameter and right ventricular outflow tract diameter.

Previous studies in in vitro using isolated vessels have indicated that activation of BK_Ca_ channel with NS1619 results in pulmonary artery (PA) dilation found in patients with pulmonary hypertension (Vang et al. 2010). To identify the role of BK_Ca_ in pulmonary arteries, we measured right ventricular outflow tract (RVOT) and PA diameter at baseline and then 1 and 15 min post injection. We interestingly did not find any significant changes in either RVOT or PA diameter (Fig. 5) as would have been expected from ex vivo work (Vang et al. 2010).

To study the functional consequence of inhibition of BK_Ca_ channels, we measured mean pulmonary velocity and mean gradient using color Doppler. PAX at both lower (2.5 ng/mL) and higher (50 ng/mL) concentrations showed a significant reduction in the mean pulmonary velocities and mean gradient (Fig. 5). These reductions in pulmonary velocities showed near complete reversibility after 15 min. IBTX, however, showed no change in mean pulmonary velocity at lower or higher concentrations even after 15 min (Fig. 5).

## Discussion

BK_Ca_ channels are ubiquitously expressed in the plasma membrane of vascular smooth muscle cells (Jia et al. 2013), cardiac fibroblasts (Li et al. 2009), and endothelial cells (Rusko et al. 1992; Ungvari et al. 2002). Surprisingly in adult cardiomyocytes, a unique BK_Ca_ channel splice variant is exclusively present in the mitochondria (Singh et al. 2012, 2013). The pore‐forming *α*‐subunit interacts with regulatory *β*‐subunits, and *β*‐subunits are also widely expressed in the heart and VSMCs (Brenner et al. 2000). Several ex vivo studies have shown that in the vascular system, BK_Ca_ channels are the key players in the regulation and maintenance of vascular tone (Ghatta et al. 2006). Interaction of *α*‐subunit with regulatory *β*‐subunits enhances the Ca^2+^ sensitivity of the BK_Ca_ channel which allows basal activity under physiological conditions of negative membrane potential of VSMCs (Tanaka et al. 1997) and possibly in mitochondrial membranes. BK_Ca_ channels are known to provide an endogenous compensatory mechanism to buffer vasoconstriction by opening in response to the depolarization of VSMCs and the rise in cytosolic Ca^2+^ concentrations (Nelson et al. 1995). However, the role of BK_Ca_ channels in cardiovascular function is marred with controversies ranging from the absence of BK_Ca_ channels at the plasma membrane on adult cardiomyocytes to whether vasodilator effect of BK_Ca_ channels in VSMCs is reduced, is persistent, or increases under pathophysiological conditions (Rusch 2009). The key reason for these discrepancies are due to focus of most of the studies on ex vivo experimental approaches such as isolated VSMCs or vessels, and complex signaling mechanisms (Singh et al. 2016) involving BK_Ca_ channels.

In a swine model, NS1619, an agonist for BK_Ca_ channels induced significantly smaller coronary arteriolar dilation in metabolic syndrome as compared with controls indicating a reduction in functional BK_Ca_ channels (Borbouse et al. 2009). In addition, changes in expression and properties of BK_Ca_ have been reported in different pathophysiological conditions, such as hypoxia, hypertension, and diabetes (England et al. 1993; Sobey 2001), indicating that BK_Ca_ plays a direct role in maintaining vascular physiology. In the heart, BK_Ca_ has been shown to play a direct role in cardioprotection from ischemia–reperfusion injury (Singh et al. 2013; Soltysinska et al. 2014; Frankenreiter et al. 2017). In sinoatrial node cells (SANCs), expression of functional BK_Ca_ channels was characterized and pharmacological inhibition by PAX in vivo was shown to be involved in cardiac pacing (Imlach et al. 2010; Lai et al. 2014). Using genetic and pharmacological tools, BK_Ca_ channels were implicated in SANCs firing rate (Lai et al. 2014). In a recent study, cardiomyocyte‐specific BK_Ca_ null mutant mice exhibited mild hypotension, a slight decrease in heart rate, as well as left ventricular ejection fraction and fractional shortening (Frankenreiter et al. 2017). This was a surprising finding as mechanisms involving baroreceptors, renal, and neuroendocrine systems in blood pressure expressed normal levels of BK_Ca_, indicating that BK_Ca_ present in cardiomyocytes causes cardiac dysfunction and blood pressure dampening.

In this study, our focus was to decipher the role of BK_Ca_ channels in the cardiovascular system in vivo. While in vitro and ex vivo investigation into the role of BK_Ca_ channels has previously been performed, in our study we were able to gain insight into in vivo function of this channel. BK_Ca_ channels inhibition had a statistically significant negative chronotropic effect on heart rate in agreement with earlier studies (Imlach et al. 2010; Lai et al. 2014). While we also believe it to possibly have a negative inotropic effect evidenced by a small reduction in LVEF and GLS (Frankenreiter et al. 2017), our results did not show statistical significance. Inhibition of BK_Ca_ channels showed a significant effect on coronary flow. Furthermore, the effects of BK_Ca_ channels inhibition extend to the right heart as well with reduced pulmonary artery velocity, VTI, and right heart stroke volume.

Bradycardia mediated by inhibition of BK_Ca_ channels is the direct result of SA nodal cell inhibition and reduction of SA nodal action potential generation (Imlach et al. 2010; Lai et al. 2014). In our study, we found that PAX treatment, but not IBTX treatment, resulted in a lower heart rate as shown earlier (Imlach et al. 2010; Lai et al. 2014). In myocardial tissue, BK_Ca_ channels have been shown to be found only intracellularly, localized to the mitochondria (Singh et al. 2013). PAX is able to cross the plasma membrane (Manzanares et al. 2011) and bind to intracellular BK_Ca_ channels in addition to plasma membrane components. IBTX does not cross the external plasma membrane (Bingham et al. 2006) and typically only binds to BK_Ca_ channels at the external plasma membrane. The fact that no persistent differences in heart rate or mean arterial pressure was seen in the DMSO group or the IBTX group confirms that the BK_Ca_ channels mediated reduction in heart rate could also be due to intracellular BK_Ca_ channels and not a vagal mediated effect or reflexive tachycardia in response to the peripheral vascular tone. The mechanisms by which BK_Ca_ channels inhibition affects myocytes is complex. Within the SA node, the inhibition of BK_Ca_ channels yields prolonged diastolic depolarization and decreased SA nodal cell excitability, resulting in bradycardia (Lai et al. 2014). Initial studies establishing that BK_Ca_ inhibition results in bradycardia showed that in addition to PAX, high‐dose IBTX slowed the heart rate in isolated rat hearts, suggestive that the target BK_Ca_ receptor is in the plasma membrane of cardiac cells (Imlach et al. 2010; Lai et al. 2014). However, the subsequent finding that BK_Ca_ is only present in the mitochondria of adult cardiac myocytes challenges this assumption (Singh et al. 2013).

Mitochondrial BK_Ca_ channels (Singh et al. 2012) like other mitochondrial ion channels regulate many intracellular activities, including ROS production, calcium accumulation, and modulation of ATP production (Ponnalagu and Singh 2017; Gururaja Rao et al. 2018). Activation of mitochondrial BK_Ca_ channels in cardiomyocytes results in reduced ROS production, increased calcium retention capacity, and delay in mitochondrial permeability transition pore opening, and inhibition of BK_Ca_ channels in isolated mitochondria has been shown to have the opposite effect (Singh et al. 2012; Toro et al. 2014; Balderas et al. 2015). Presumably, it is the effect of calcium accumulation which leads to delayed depolarization. Furthermore, differential expression of the *β* subunit may also be a factor in the effects exerted by various drugs in different tissues. Out of four known *β* subunits, cardiac tissue contains predominantly the *β*3 and *β*4 subunits (Li and Yan 2016). Presence of the *β*4 subunit confers resistance to IBTX (Meera et al. 2000), and its presence in cardiac myocytes may explain why PAX but not IBTX showed a significant effect in our study. A third mechanism that may contribute to the bradycardic effect of PAX but not IBTX is the central nervous system (CNS) effect of PAX – neurons within the CNS preferentially express the *β*4 subunit and are very resistant to the effects of IBTX (Wang et al. 2014). BK_Ca_ inhibition of the sympathetic nerve cells results in prolonged depolarization and decreased action potential firing, resulting in decreased sympathetic innervation to the heart and decreased heart rate.

In addition, it is possible that extracardiac BK_Ca_ channels within cardiac neurons can play a role in heart rate modulation and this may explain why some studies have seen an effect with IBTX. Cardiac neurons have been shown to play a role in mediating vasomotor tone in response to electromechanical forces within the ventricle and function to preserve heart rate and ventricular contractility (Arora et al. 2001). They are both PAX and IBTX sensitive and have already been demonstrated to have effects in cardiac ischemia–reperfusion injury (Scornik et al. 2001; Perez et al. 2013; Wojtovich et al. 2013). Although our study was supportive of the effects of BK_Ca_ inhibition being mediated wholly by intracardiac mitochondrial BK_Ca_, further investigation is needed to discern the role of both populations of BK_Ca_ in cells.

Expression of BK_Ca_ channels in coronary arteries diminishes with age in rats and human beings without affecting the biophysical or pharmacological properties (Marijic et al. 2001). In contrast, exercise ameliorates expression of BK_Ca_ channels in coronary arteries in aged rats (Albarwani et al. 2010). In agreement with a reduction in expression of BK_Ca_ channels, a significant reduction was reported in contraction capacity in coronary arteries of aged rats in ex vivo experiments (Marijic et al. 2001). Our results for the first time show that inhibition of BK_Ca_ channels plays a significant role in maintaining coronary function. Coronary blood flow exhibits a typical dose–response curve to increasing heart rate. Blood flow per single cardiac cycle, however, is reduced at increased heart rate, reflecting the decrease in diastolic duration. Thus, the bradycardia incurred from PAX is not a contributing factor to our decrease in coronary flow, but rather an impairment of BK_Ca_ channels mediated vasodilation.

Even though the role of BK_Ca_ channels in cardiac mitochondria in cardioprotection from ischemia–reperfusion injury has been demonstrated by independent groups (Singh et al. 2012, 2013; Soltysinska et al. 2014; Frankenreiter et al. 2017), at least one group has indicated the role of noncardiomyocyte BK_Ca_ channels in cardioprotection (Wojtovich et al. 2013). A recent study has also shown that BK_Ca_ channels present in adult cardiomyocytes play a direct role in cardioprotection by using cardiomyocyte‐specific knockout mice (Frankenreiter et al. 2017). However, the role of BK_Ca_ channels present in other cells and organs could also be partially contributing to the cardioprotection and cardiac function. Regulation of BK_Ca_ channels in maintaining the coronary artery flow may also support the finding of reduced infarct size in animal models that undergo BK_Ca_ channels—activation prior to or immediately after an ischemic injury (Singh et al. 2013). A reduction in blood flow is well‐characterized to slow oxygen delivery which may limit production of free radicals in the cardiac tissue. Increase in free radicals which also results in Ca^2+^ overload mediated by blocking BK_Ca_ channels are well‐characterized and established factors for the cardiac ischemia–reperfusion injury (Stowe et al. 2006; Heinen et al. 2007; Aldakkak et al. 2008; Singh et al. 2012, 2013).

Evaluation of the cardiac function was performed by echocardiography, and global longitudinal strain analysis was carried out. Although this study represents a significant step forward in elucidating the physiologic effects of pharmacologic regulation of BK_Ca_, there are some limitations to the usage of echocardiography. Echocardiography is inherently subject to some variability in interpretation. To minimize variability in interpretation, quantitative measurements were used, computed by delineation of the endocardial border. Our quantitative measures of LV function did not show any statistically significant data, although qualitative changes in wall motion were seen. However, we chose not to report specific details of qualitative measures like wall motion abnormality (WMA) due to the high degree of interobserver variability when judging their severity and significance. Moreover, WMAs are generally consequences of decreased coronary perfusion and flow, parameters which were measured quantitatively and found to be reduced after exposure to PAX and IBTX.

Our LVEF and strain data sets did not bear out statistical significance, and strain analysis showed cardiac dysfunction with PAX which was reversible after 15 min of injection. Though LVEF is the most commonly used marker to assess for LV dysfunction, there are some limitations to its use in cardiac studies. Strain analysis allows for more sensitive evaluation of myocardial contractility and can detect differences in LV function that may be missed when using LVEF alone. Another advantage is that strain analysis can be performed retroactively during postimaging analysis as long as high‐quality images have been captured using a standard imaging protocol. We also evaluated the role of BK_Ca_ channels on the right heart as the right side of the heart in the context of BK_Ca_ channels is not extensively studied. In terms of right heart, we noted a statistically significant decrease in pulmonary flow velocity and mean pulmonary gradient. These changes appear to occur independently of vascular tone as we did not witness a significant change in RVOT or PA diameter. This would suggest that the decreases in flow were an extension of a decrease in cardiac output driven primarily by PAX‐induced bradycardia.

Given the widespread cardiac effects of BK_Ca_ channels inhibition observed, the translation ability of these findings is promising. However, the most significant and consistently observed reversible effect of PAX is sinus bradycardia. It seems that many of the cardiac effects we observed were likely related to the BK_Ca_ channels in SNACs and coronary arteries. This opens the door for BK_Ca_ channels blockade as a means of antiarrhythmic control in patients who do not tolerate beta blockers or other classes of heart rate‐controlling medications (Zimetbaum 2012; Maan et al. 2013). An optimal dose of BK_Ca_ channels blocker/inhibitor such as PAX could therapeutically lower heart rate without significantly affecting other cardiac variables could prove useful. The most significant finding is that this impact on BK_Ca_ channels is completely reversible. In summary, using pharmacological tools, we have performed in vivo comprehensive evaluation and showed the role of BK_Ca_ channels on cardiovascular function.

## Conflict of Interest

Authors have no conflicts of interest to disclose.
